# B Cell Responses to HIV Antigen Are a Potent Correlate of Viremia in HIV-1 Infection and Improve with PD-1 Blockade

**DOI:** 10.1371/journal.pone.0084185

**Published:** 2013-12-16

**Authors:** Katherine J. Nicholas, Emily K. Zern, Louise Barnett, Rita M. Smith, Shelly L. Lorey, Courtney A. Copeland, Shanmugalakshmi Sadagopal, Spyros A. Kalams

**Affiliations:** 1 Department of Pathology, Microbiology and Immunology, Department of Medicine, Vanderbilt University School of Medicine, Nashville, Tennessee, United States of America; 2 Division of Infectious Diseases, Vanderbilt University School of Medicine, Nashville, Tennessee, United States of America; 3 Department of Molecular Physiology and Biophysics, Vanderbilt University School of Medicine, Nashville, Tennessee, United States of America; Karolinska Institutet, Sweden

## Abstract

Infection with Human Immunodeficiency Virus Type 1 (HIV-1) induces defects of both cellular and humoral immune responses. Impaired CD4+ T cell help and B cell dysfunction may partially explain the low frequency of broadly neutralizing antibodies in HIV-infected individuals. To understand the extent of B cell dysfunction during HIV infection, we assessed the level of B cell activation at baseline and after stimulation with a variety of antigens. Increased levels of viremia were associated with higher baseline expression of the activation marker CD86 on B cells and with decreased ability of B cells to increase expression of CD86 after *in vitro* stimulation with inactivated HIV-1. In a series of cell isolation experiments B cell responses to antigen were enhanced in the presence of autologous CD4+ T cells. HIV infected individuals had a higher frequency of PD-1 expression on B cells compared to HIV- subjects and PD-1 blockade improved B cell responsiveness to HIV antigen, suggesting that inhibitory molecule expression during HIV-1 infection may contribute to some of the observed B cell defects. Our findings demonstrate that during chronic HIV infection, B cells are activated and lose full capacity to respond to antigen, but suppression of inhibitory pressures as well as a robust CD4+ T cell response may help preserve B cell function.

## Introduction

Infection with HIV-1 induces defects of both cellular and humoral immune responses, inhibiting the immune system from mounting an effective response against infection. Since shortly after AIDS was identified, abnormalities of both B cell and T cell function have been described in HIV-infected individuals [1]. Persistent high level viremia is associated with increased expression of activation markers on T and B cells [2,3], hypergammaglobulinemia [1,4-6], and decreased antibody responses to *in vivo* vaccination [7-10]. In addition to antibody production, B cell antigen presenting function is also impaired after HIV infection [11]. While it has been suggested that B cell function may be deficient as a result of a lack of CD4+ T cell help [12], there also may be intrinsic B cell defects in HIV infection [13].

B cells in chronic viral infection have a phenotype consistent with immune exhaustion and terminal differentiation [14-16]. In HIV-infected individuals, expression of the IL-2 receptor, CD25, on B cells in response to *in vitro* stimulation is lower than in uninfected individuals, despite normal levels of expression of CD154 (CD40L) on CD4+ T cells. This defect persists even after the addition of supplemental IL-2 [13]. The bidirectional interaction between CD80 and CD86, ligands of the B7 family, and their receptor, CD28 on CD4+ T cells, is also critical for an effective humoral response. In HIV infection, B cells of viremic subjects not only have decreased ability to increase expression of CD80 and CD86 in response to *in vitro* BCR and CD40L stimulation, but they also are ineffective at stimulating CD4+ T cells, suggesting impairment in both directions of the interaction [17]. 

The decreased responsiveness of B cells may be due to impaired help they receive from exhausted CD4+ T helper cells in HIV infection [18-21]. Exhausted CD4 and CD8 T cells exhibit decreased responses to antigen and often express high levels of inhibitory receptors such as PD-1 and CTLA-4 on their surface. Studies have likewise termed B cells “exhausted” due to their poor proliferative capacity that is only partially restored with the addition of stimulatory cytokines and soluble CD40L [14,16]. Increased surface expression of PD-1 on T cells is sustained over the course of chronic viral infection [22,23] and may define a reversible impairment of HIV-specific T cell function [18-20,24,25]. The function of T cells from HIV-infected individuals can be partially restored by *in vitro* blockade of the PD-1/PD-L1 interaction [18,26,27]. After acute SIV infection, *in vivo* blockade of PD-1 has been shown to increase the proliferative capacity and frequency of B cells and the production of SIV-specific binding antibody [28]. B cells from HIV-infected individuals have increased expression of several inhibitory receptors, and siRNA downregulation of these receptors increases memory B cell proliferation and increases the number of antibody-secreting B cells [29]. While blocking these inhibitory pathways may provide opportunities to restore CD4+ T cell help for B cells, these interactions have not yet been directly evaluated.

We measured B cell activation markers CD25 and CD86 in the setting of chronic HIV-1 infection after *in vitro* culture with and without stimulation of PBMCs by a variety of antigens. We found high frequencies of CD86+ B cells in HIV-infected individuals, and their frequency correlated with the level of viremia. B cell responsiveness to inactivated HIV, however, negatively correlated with viral load. We also performed a series of co-culture experiments with purified B cells and autologous CD4+ T cells, as well as *in vitro* blockade of PD-1 to investigate the requirements for CD4+ T cell help and the role of inhibitory molecules for inducing B cell activation. We provide evidence that lack of HIV-specific CD4 helper responses and high PD-1 expression in the setting of HIV-1 infection both contribute to B cell dysfunction.

## Materials and Methods

### Study Subjects

 Study subjects included seven HIV-negative controls and 21 HIV-infected individuals (Table 1). PBMCs were separated from blood samples using a Ficoll-Paque^TM^ Plus density gradient, cryopreserved in FBS with 10% DMSO, and stored in liquid nitrogen until thawed for immediate use. HIV-infected subjects were ART-naïve with wide ranges of CD4 T cell numbers (132 to 1374 cells/uL), viral load (50 to 76,427 copies/mL), and duration of infection (<1 to 23 years post-diagnosis). The Vanderbilt University School of Medicine IRB approved this study, and all individuals provided written informed consent. All HIV-infected individuals were recruited from the Vanderbilt Comprehensive Care Center, and HIV negative individuals were recruited from Vanderbilt University Medical Center.

**Table 1:** HIV+ subject characteristics.

	**Subject ID **	**Time post-infection (years)**	**Viral Load (copies/mL)**	**CD4+ T cells (per mm^3^ blood)**
**1**	10031	11	50	856
**2**	10071	14	50	950
**3**	10040	14	50	1,161
**4**	10067	17	199	814
**5**	10004	19	253	360
**6**	10060	4	722	688
**7**	10070	12	750	915
**8**	20018	2	1,886	1,374
**9**	20011	<1	2,655	512
**10**	10023	6	7,178	390
**11**	10027	14	7,340	378
**12**	10054	3	7,522	350
**13**	10035	20	7,750	464
**14**	10026	6	8,865	414
**15**	10108	3	9,698	960
**16**	20028	<1	15,800	515
**17**	10042	23	20,211	331
**18**	10114	17	20,574	966
**19**	10076	6	21,339	700
**20**	20017	1	23,317	435
**21**	10086	17	76,427	132

Organized by increasing viral load.

### *In vitro* cell stimulation

 Peripheral blood mononuclear cells (PBMCs) from each subject were cultured at 10 x10^6^ cells/mL in 48-well plates (2x10^6^ cells/well) in R10 medium (RPMI 1640 containing 10% heat inactivated FCS, 2 mM L-glutamine, 50 ug/mL penicillin, 50 ug/mL streptomycin, and 10mM Hepes) and co-stimulated with anti-CD28 (1ug/mL, BD Biosciences) and anti-CD49d (1ug/mL, BD). Cells were non-specifically stimulated with plate-bound OKT3 (1 ug/ml, ATCC) or Staphylococcal Enterotoxin B (SEB) (1ug/mL, Sigma). HIV-specific stimulation was performed with AT-2 inactivated HIV-1 MN particles (0.53ug/mL p24, Lot# P3964, generously provided by Dr. Jeff Lifson) [30,31], or HIV-1 p24 core protein (1ug/mL, Protein Sciences). As controls, PBMCs were incubated with media alone, or HIV-1 MN control, containing AT-2 treated microvesicles prepared from matched uninfected cultures, used at a comparable total protein concentration (Lot# P3914) [30,31]. At 24 hours post-stimulation, PBMCs were recovered, washed, and stained with the appropriate surface marker antibodies. For experiments evaluating PD-1 blockade, PBMCs were incubated overnight with 10ug/ml anti-PD-1 (EH12.2H7, BioLegend).

### Flow cytometric evaluation of lymphocyte surface molecules

 B and T cell surface markers were analyzed by flow cytometry after 24 hours of stimulation using a combination of an amine-reactive viability dye (LIVE/DEAD Aqua, Invitrogen), CD3-AF700 (UCHT1, BD), CD4-PETR (S3.5, Invitrogen), CD8-V450 (RPA-T8, BD), CD19-PECy-7 (SJ25C1, BD), CD25-FITC (2A3, BD), CD86-PE (2331, BD) and PD-1-PE (EH12.2H7, BioLegend). In prior studies, we used an indirect staining method for detection of PD-1 with purified anti-PD-1 (Mouse IgG1, clone EH12.2H7, BioLegend) followed by goat-anti-mouse IgG-Pacific Blue (Molecular Probes) [32,33]. However, this lead to very high background staining of CD19+ B cells (i.e. the goat anti-mouse antibody labeled B cells even in the absence of a primary antibody) that could not be overcome by incubation of cells with human serum. The anti-PD-1-PE directly conjugated antibody yielded frequencies of PD-1 expression on CD4+ T cells similar to the indirect method. CD4v4-PE (L120, BD) was used to stain CD4 cells after they had been purified using methods described below. Lymphocytes were discriminated based on cell size and granularity using forward and side-scatter properties. Non-viable cells were gated out of further analysis. CD19+ B cells were evaluated for surface expression of activation (CD25 and CD86) markers. CD4+ T cells were selected from their parent CD3+ T cell population and evaluated for CD25 expression.

### *In vitro* stimulation after B cell and CD4+ T cell isolation

 CD19+ B cells were purified using EasySep Human CD19 Positive Selection Kit and Robosep (StemCell Technologies). CD4+ T cells were purified from the CD19-depleted fraction using EasySep Human CD4 Positive Selection Kit and Robosep (StemCell Technologies). Purity was assessed after each selection step by flow cytometry: B cell purity ranged from 92-99% of live cells and CD4+ cell purity ranged from 70-90% (depending on the subject’s monocyte population). Total PBMCs, positively selected B cells, or positively selected B cells combined with positively selected CD4+ T cells were cultured overnight in a 48-well plate. Cells were left unstimulated or were stimulated with p24 antigen or SEB. As a positive control for B cell stimulation, a subset of cells from each condition was stimulated with soluble CD40L (10ng/mL, GIBCO) and purified anti-human IgM (10ug/mL, BioLegend). After 24 hours cells were washed and stained with the appropriate surface marker antibodies.

### Statistical Analysis

 All statistical analysis was done using GraphPad Software (Prism 6). Correlations were performed using the Spearman rank method. All paired comparisons were analyzed with the Wilcoxon matched pairs t-test while unpaired comparisons were analyzed using the Mann Whitney t-test.

## Results

### B cell responsiveness correlates with HIV viremia

**Figure 1:**
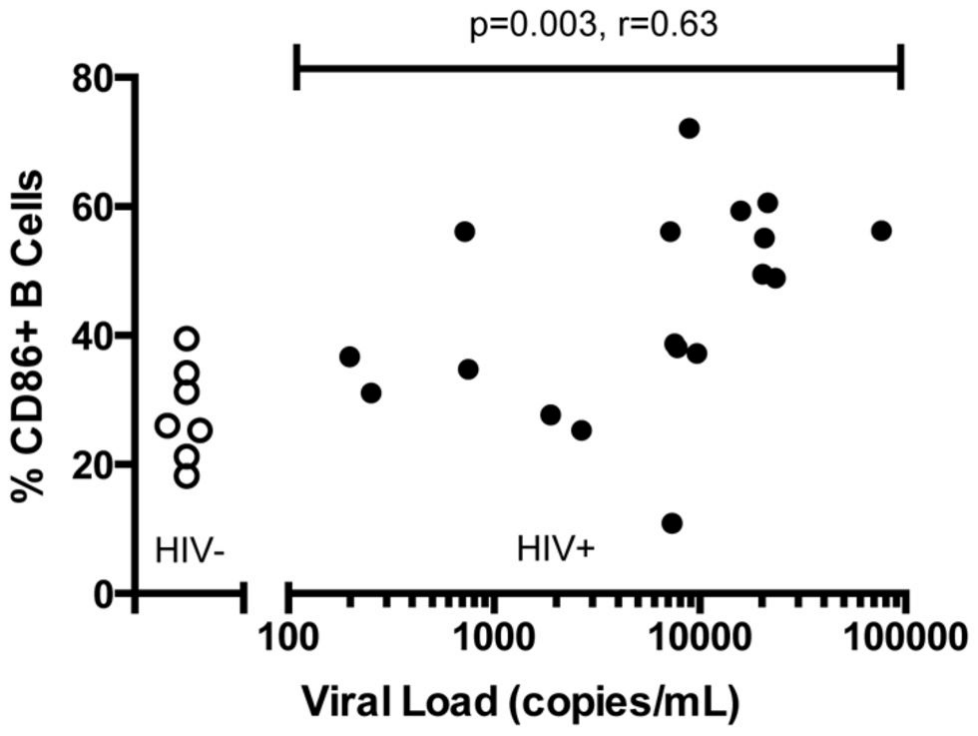
CD86+ B cells are more frequent in HIV+ than HIV- subjects and correlate with viremia. PBMCs from HIV- (open circles) or HIV+ (closed circles) subjects were incubated overnight without stimulation and evaluated for surface level CD86 expression on B cells. HIV+ subjects had a higher frequency of CD86+ B cells compared to HIV- subjects (unpaired t-test not shown on graph, p=0.03). The frequency of CD19+CD86+ B cells in HIV+ individuals correlates with the level of viremia (r=.63; p=.003). Correlation statistics shown are derived from HIV+ subject data only and do not include data from HIV- subjects.

We first measured the baseline expression of the activation markers CD86 and CD25 on B cells from a cohort of HIV-infected individuals with a wide range of viremia (Table 1) and seven HIV-negative controls. We found that the baseline frequency of CD86-expressing B cells in HIV-infected individuals was higher than that of uninfected control subjects (medians of 38% compared to 26%, p=0.03) and positively correlated with viral load (Figure 1; r=0.63, p=0.003). Baseline expression of CD25 on B cells (p=0.48) or CD4+ T cells (p=0.97) did not correlate with viral load. 

**Figure 2:**
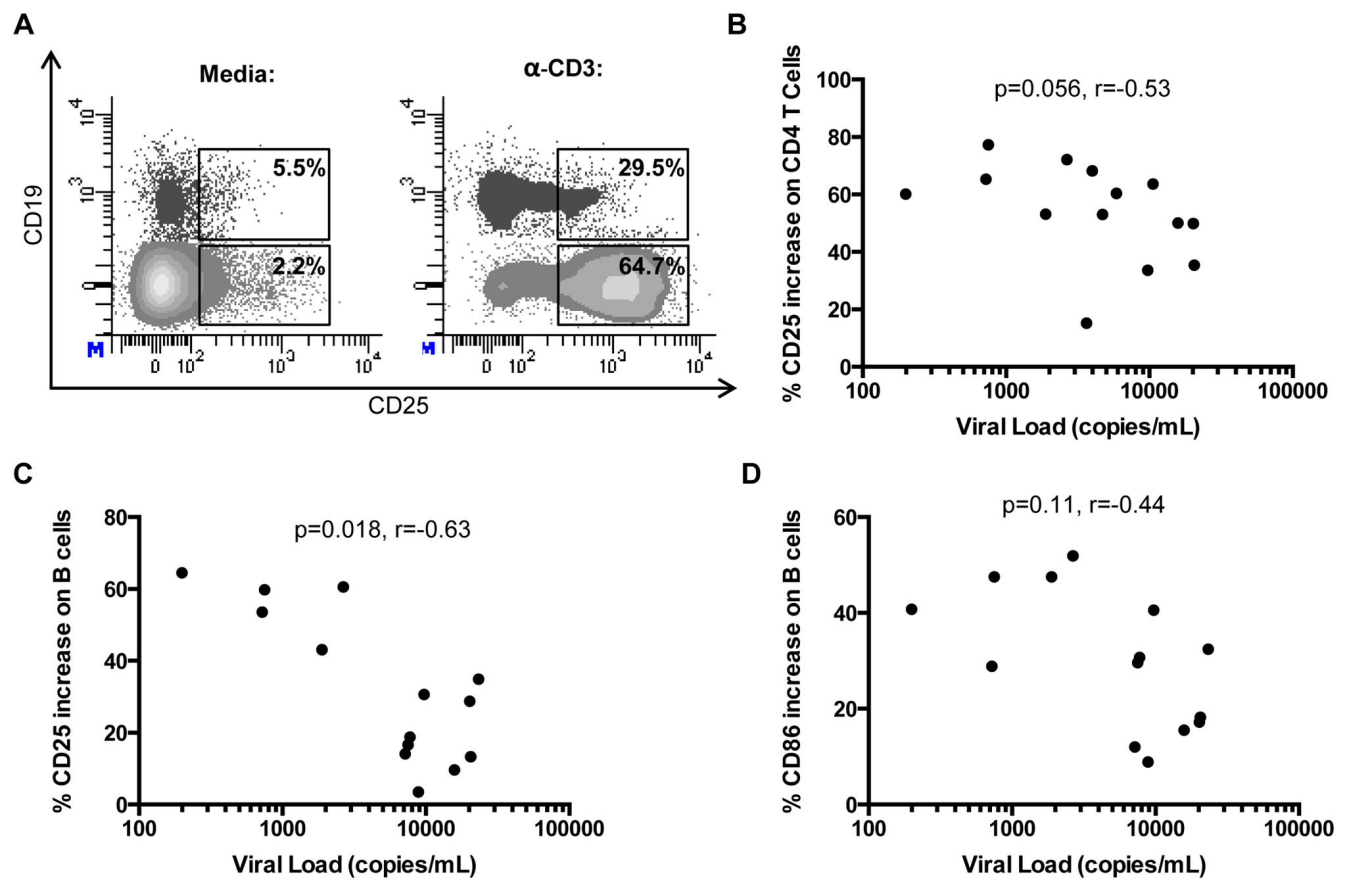
Change in CD25 expression on B and CD4+ T cells negatively correlates with viral load. PBMCs were cultured overnight with or without anti-CD3 stimulation. Change in CD25 or CD86 expression was determined by subtracting the frequency of expression before stimulation from the frequency of expression after stimulation. (**A**) Representative plots of CD25 expression on CD4+ T cells and B cells with (bottom) and without (top) anti-CD3 stimulation. CD4+ T cell population shown is CD3+CD4+CD19- and B cell population shown is CD3-CD4-CD19+. (**B**-**D**) Change in expression of CD25 on CD4+ T cells (r= -.53; p= .056) (B), CD25 on B cells (r= -.63; p= .018) (C), and CD86 on B cells (r= -.44; p= .11) (D) correlates negatively with viral load.

It has previously been shown that B cells from HIV-infected individuals have a diminished ability to express CD25 after *in vitro* stimulation, and this contributes to low proliferative capacity [13]. We measured the expression of CD25 on both CD4+ T cells and B cells after direct stimulation of PBMCs with anti-CD3 antibody in 14 HIV-infected individuals off anti-retroviral therapy with a range of viral loads (Figure 2A). CD25 expression on CD4+ T cells increased after anti-CD3 stimulation, and there was a weak negative correlation between the change in expression of CD25 and viral load (Figure 2B, r=-0.53, p=0.056). There was a strong inverse correlation between the change in expression of CD25 on B cells (Figure 2C, r=-0.63, p=0.02) and viral load. The change in expression of CD86 on B cells in response to anti-CD3 did not correlate with viral load (Figure 2D, r=-0.44, p=0.11).

**Figure 3:**
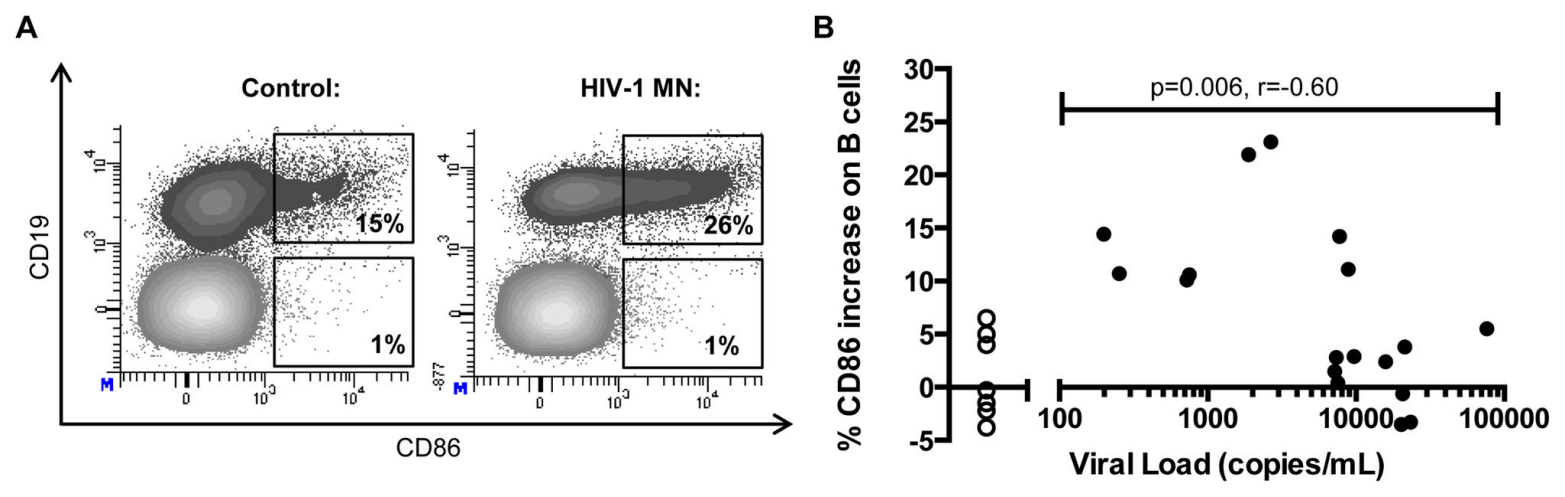
Magnitude of B cell responses to inactivated HIV-1 inversely correlates with viral load. (**A**) Expression of CD86 on B cells after stimulation of PBMC with HIV-1 MN control (left column) or HIV-1 MN (right column). Shown are representative plots from one individual (subject 10071).. (**B**) PBMCs from 21 HIV-infected individuals (closed circles) and 7 HIV-negative control subjects (open circles) were incubated with inactivated HIV-1 MN, and changes in CD86 expression on B cells were measured. Change was calculated by subtracting the frequency of CD86 expression from stimulation with the HIV-1 MN control (containing no HIV proteins) from stimulation with HIV-1 MN. CD86 expression on B cells in response to HIV-antigen in HIV infected individuals is negatively correlated with viral load (r= -.6; p= .006). Correlation statistics are only applied to HIV-infected individuals and do not include data from uninfected subjects.

To measure antigen-specific responses, we next evaluated the ability of B cells to respond to *in vitro* stimulation with aldrithiol-2 (AT-2) inactivated HIV-1 MN in the presence of CD4+ T cells. Bidirectional activation of B cells and CD4+ T cells requires co-stimulatory interactions between CD80/CD86 on B cells and CD28 on CD4+ T cells. We stimulated PBMC with AT-2 inactivated HIV-1 MN or HIV-1 MN control (Figure 3A). Responses to treatment by HIV-1 MN control were not above baseline levels. In HIV-negative control subjects, we observed minor differences between expression of CD86 on B cells by inactivated HIV antigen compared to the control (median of -0.3%). However, HIV-infected individuals responded to HIV antigen, and the degree of CD86 expression was inversely correlated with the level of viremia (median of 4.95%, r=-0.60, p=0.006) (Figure 3B). Responsiveness of CD4+ T Cells to HIV-1 MN as measured by increased expression of CD25 was significantly higher in HIV infected individuals compared to negative controls (p=.02), but the degree of responsiveness to HIV antigen did not correlate with viral load (p=0.4, data not shown). These results demonstrate that in chronic HIV-1 infection, the activation state and responsiveness of B cells to both HIV and non-HIV antigens are much better correlates of control of viremia than similar parameters on CD4+ T cells. 

### Autologous CD4+ T cells enhance B cell responses to HIV antigen

**Figure 4:**
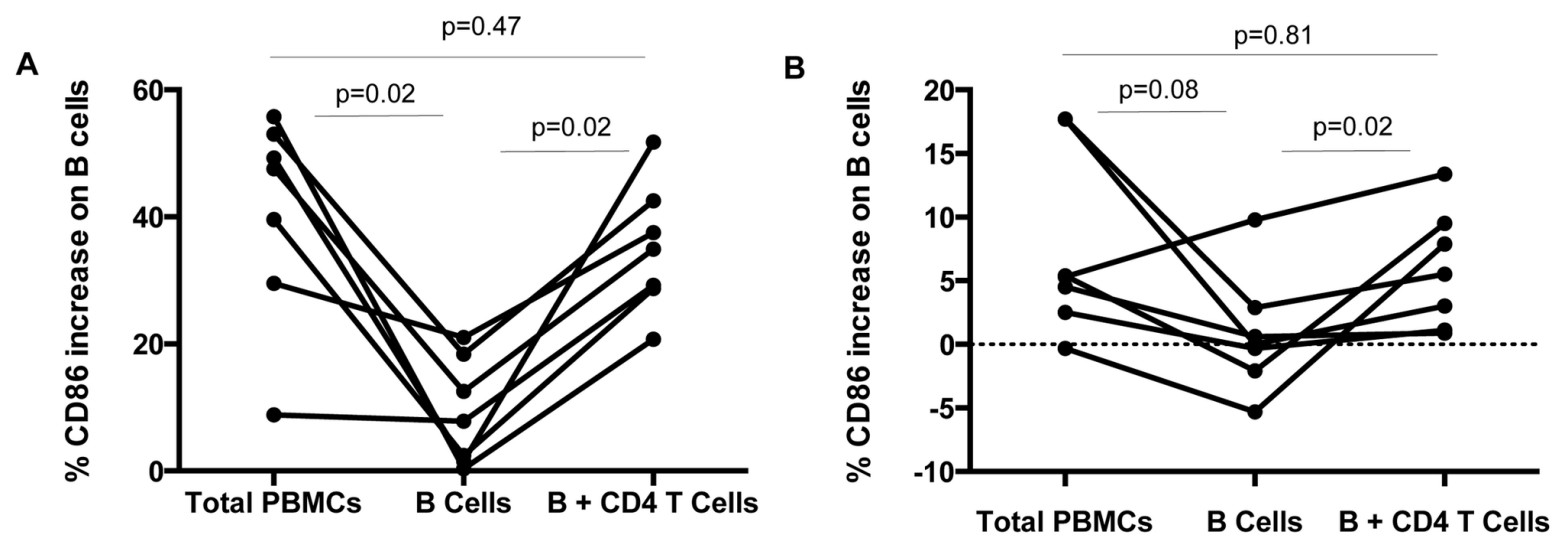
Purified B cell responses to p24 are enhanced by addition of autologous CD4+ T cells. Change in the frequency of CD86+ B cells in response to SEB (A) or HIV p24 antigen (B) was evaluated in total PBMC culture, purified B cell culture, or purified B cells co-cultured with autologous purified CD4+ T cells.

We next evaluated the contribution of CD4+ T cells for B cell responses to antigenic stimulation. We purified B cells from 7 HIV-infected individuals who demonstrated responses to HIV antigen and who had sufficient numbers of PBMCs available for analysis, and measured CD86 expression after *in vitro* stimulation in the presence or absence of CD4+ T cells. Previous studies have demonstrated that HIV-specific CD4+ helper responses are primarily Gag-specific [26,34]. In our lab we observed that CD4+ T cells respond more robustly to p24 protein stimulation compared to inactivated HIV-1 MN stimulation (data not shown). Since we sought to measure the CD4+ T cell contribution to B cell activation, in this series of cell isolation experiments we stimulated cultures with HIV p24 antigen. We found that in the absence of CD4+ T cells, purified B cell populations had a markedly reduced ability to respond to the superantigen SEB (Figure 4A; p= .02) or HIV p24 antigen (Figure 4B; p= .02). The ability of B cells to respond to antigen was restored after addition of autologous CD4+ T cells back to the cultures. As a positive control, B cells were able to directly respond to anti-IgM and soluble CD40L to a similar extent whether CD4+ T cells were present or not (data not shown). These data suggest that in this system, B cells have an enhanced response to HIV antigen when they receive help from antigen-specific CD4+ T cells. 

### PD-1 blockade improves B cell responses to HIV antigen

**Figure 5:**
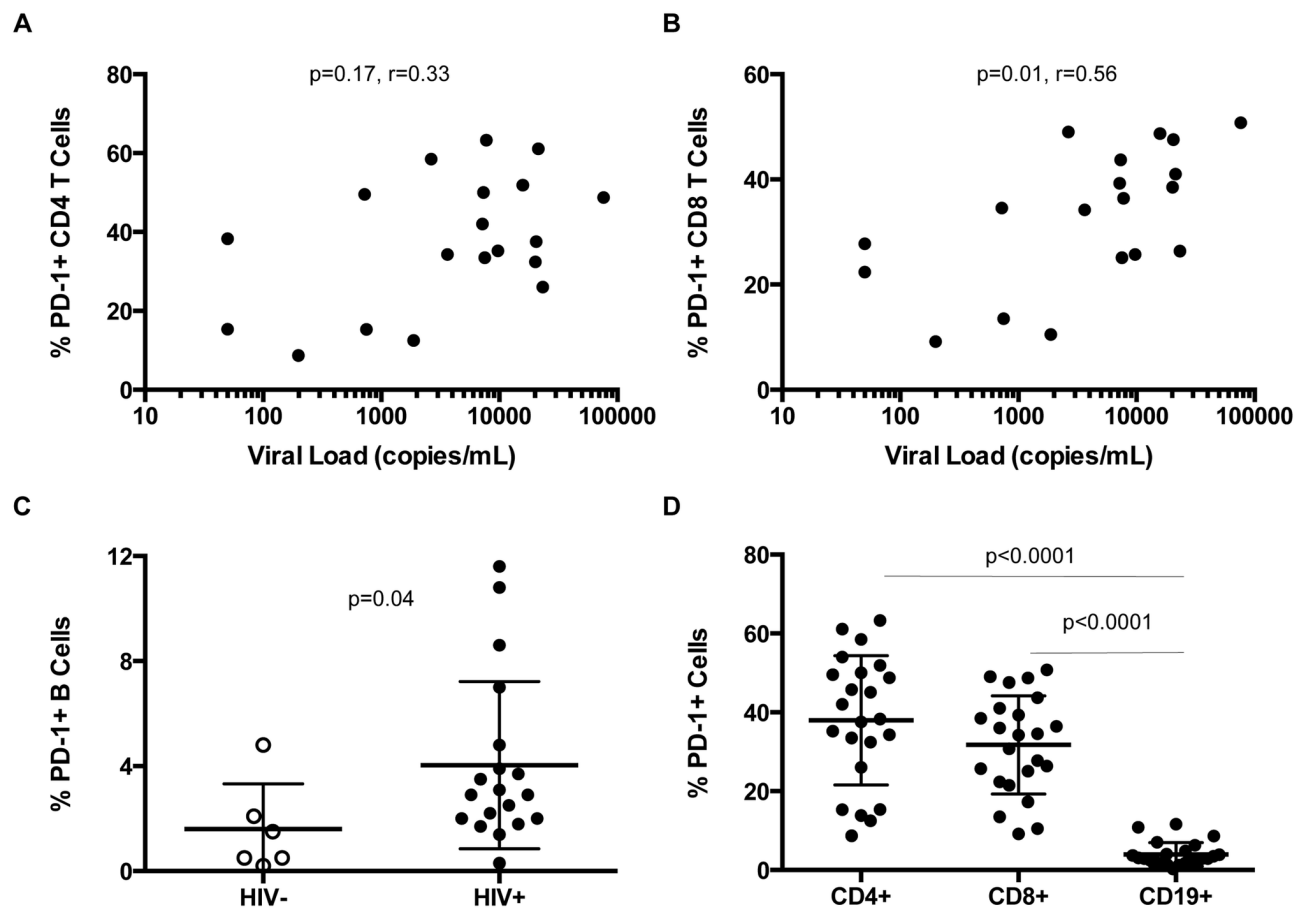
Frequency of PD-1 surface expression on lymphocytes is elevated during HIV infection. Expression of PD-1 on B cells, CD4+ T cells, and CD8+ cells was measured directly *ex* *vivo*. (**A**) In HIV-infected individuals, PD-1 expression on CD4+ is not correlated with viral load (r=.33; p=.17). (B) In HIV-infected individuals PD-1 expression on CD8+ T cells correlated positively with viral load (r= .56; p=.01). (**C**) PD-1 expression is higher on B cells from HIV+ (closed circles) compared to HIV- (open circles) subjects (p=.04). (**D**) The frequency of PD-1 surface expression is significantly lower on B cells compared to CD4 (p< .0001) and CD8 T cells (p<.0001) in HIV infection.

 We next investigated whether PD-1 expression on B or T cells was related to the ability of B cells to express CD86 in response to *in vitro* stimulation. We measured surface PD-1 expression on T cells and B cells in our cohort; there was no correlation between frequencies of PD-1-expressing CD4+ T cells and viremia (Figure 5A; r=.33, p=.17), but higher frequencies of PD-1-expressing CD8+ T cells were associated with increasing levels of viremia (Figure 5B; r=0.56, p=0.01). While the frequency of PD-1+ CD4+ T cells (p = .06) and B cells (Figure 5C; p=0.04) was higher in HIV+ compared to HIV- individuals, PD-1 expression on these cell subsets did not correlate with viral load. The PD-1 expression on CD19+ B cells was significantly lower than that of CD8+ T cells and CD4+ T cells for all individuals studied (Figure 5D; p<0.0001). 

**Figure 6:**
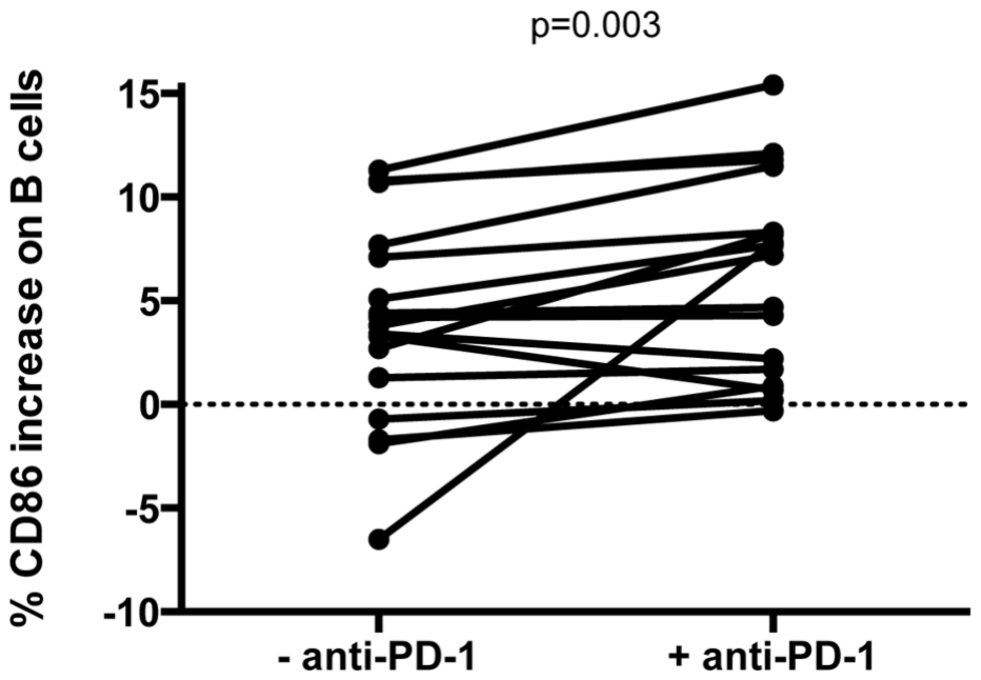
PD-1 blockade improves B cell responses to stimulation with inactivated HIV-1. PBMCs were cultured overnight with or without anti-PD-1 and stimulated with inactivated HIV-1 MN protein. Change in response to MN stimulation was calculated by subtracting stimulation with MN control protein from stimulation with HIV-1 MN protein (p= .003).

To determine whether lymphocyte PD-1 expression affects B cell stimulation, we evaluated the effect of PD-1 blockade on *in vitro* B cell activation in response to HIV antigen. We observed that increased CD86 expression on B cells in response to inactivated HIV-1 MN was enhanced by *in vitro* PD-1 blockade in HIV-infected individuals (Figure 6; p=0.003). However, while statistically significant, the median increase in CD86 expression in the presence of anti-PD-1 was modest (median absolute increase of 1.4%; median relative increase of 36%). We found no correlations between the effect of PD-1 blockade and viral load, baseline expression of CD86 on B cells, or baseline expression of PD-1 on CD4+ T cells or B cells. In summary, these data suggest that the elevated PD-1 expression on B cells or CD4+ T cells in chronic HIV infection may contribute to impaired B cell responsiveness.

## Discussion

HIV replication causes B cell hyperactivation, susceptibility to apoptosis, and poor proliferative capacity. This dysfunction in HIV-infected individuals is characterized by hypergammaglobulinemia and deficient antibody responses to both neo and recall antigens [7-10,35-38]. Here we confirm prior studies that the degree of B cell activation, as measured by the frequency of CD86-expressing B cells, is not only higher in HIV+ individuals [11,39], but also correlates with the level of viremia. We also extend these studies by demonstrating that the ability of B cells to respond to HIV antigen is a strong correlate of control of viremia.

Few studies have evaluated the relationship between B cell responses to antigen and viral load. The most consistent correlates of control of viremia are the ability of CD4+ T cells [34,40] and CD8+ T cells [41] to proliferate for several days in culture in response to HIV antigen. The frequency of HIV-specific interferon gamma-producing T cells has not been shown to correlate with control of viral replication [42], but the frequency of T cells able to secrete multiple cytokines and exert effector function is associated with proliferation following antigen exposure, and thus may be a better correlate of immune control of viremia. However, significant frequencies of polyfunctional T cells are typically observed in subjects with viral loads <1,000 copies/mL [43,44]. While we did not perform assays for cytokine responses to HIV stimulation in this study, we measured changes in CD25 expression on CD4+ T cells after stimulation with inactivated HIV-1 and the ability of B cells to respond to HIV antigen was a better correlate of viremia 

B cell activation has been linked to decreased B cell responsiveness to stimulation and decreased proliferative capacity [11,45]. Moir et. al. found that after T cell stimulation with anti-CD3, the ability of B cells to express CD25 was diminished with increasing viral load, but a strict correlation was not observed [13]. We confirmed this finding in our cohort, but also found CD86 expression on B cells in response to inactivated HIV-1 to be a strong negative correlate of control of viremia. Our study was not designed to determine which regions of the virus were targeted by these responses. AT-2 inactivated HIV-1 MN contains structurally intact viral proteins, and while we suspect these B cell responses are directed against Env, we can’t rule out recognition of other structural proteins (e.g. Gag) that are exposed during the inactivation process. Nevertheless, B cell responsiveness to HIV antigen is potentially an even stronger negative correlate of control of viremia than the frequency of virus-specific T cell responses. 

 We next evaluated whether the lack of CD4+ T cell help contributes to decreased B cell responsiveness. Initially, we performed stimulations after depleting CD4+ T cells by negative selection, but found considerable B cell responses to HIV antigen. This raised the possibility that either B cells were directly responding to antigen, or antigen presenting cells remaining in culture were providing significant help (data not shown). Repeat experiments were performed with positively selected B cells with or without the addition of autologous CD4+ T cells. B cells alone had a limited ability to respond to the T cell stimulant SEB, and this ability to respond to antigen was reconstituted with the addition of CD4+ T cells. Isolated B cells also had a diminished response to p24 stimulation, which likewise was restored by the addition of CD4+ T cells to the culture. This isolation procedure had no effect on the ability of B cells to respond directly to stimulation with anti-IgM and CD40L. We also ensured that our isolation methods were not altering the activation state of the B cells by comparing the difference in CD86 expression on B cells before and after isolation. While we cannot rule out the possibility that some of the B cell activation we observed was due to the direct recognition of HIV antigen by B cells, the results of our B and T cell isolation experiments suggest that antigen-specific CD4+ T cell help enhances B cell activation. 

To investigate a potential mechanism for poor responses of B and T cells in HIV infection, we evaluated the role of immune exhaustion through expression of PD-1 on these cell populations. In accordance with prior studies, we found that while PD-1 expression on CD8+ T cells correlated with the level of viremia, there was a weaker relationship between PD-1 expression of CD4+ T cells and the level of viremia [18-20]. PD-1 expression on B cells was much lower than that of CD4+ and CD8+ T cells, but higher on B cells from HIV-infected compared to HIV-uninfected individuals. Boliar et. al. recently reported a correlation between the frequency of PD-1+ B cells and viral load (7), but this relationship was driven by individuals with CD4+ T cell counts <200/mm^3^. We did not see this correlation in our study cohort, likely because all but one of our study subjects had CD4+ T cell counts >300/mm^3^. 

*In vitro* PD-1 blockade of PBMC from HIV-infected individuals has been shown to augment HIV-specific CD4+ and CD8+ T cell function (23). We found *in vitro* PD-1 blockade to modestly increase the ability of B cells to respond to HIV antigen. These experiments do not explain whether decreased B cell responsiveness to stimulation reflects increased PD-1 expression on B cells, CD4+ T cells, or both. However, since these interactions are bi-directional it is reasonable to assume increased PD-1 expression on both subsets of cells play a role in reducing B cell activation. Furthermore, we did not find a direct relationship between the frequency of PD-1 high CD4+ T cells or B cells, and the ability of PD-1 blockade to increase the magnitude of B cell responses. We did not investigate other pathways, such as CTLA-4, Tim-3 or LAG-3 [46-48] that may also contribute to immune exhaustion, but our results demonstrate reversal of immune exhaustion may be one of many modalities that could serve as a potential immunotherapy to help restore CD4-mediated B cell help.

We simultaneously evaluated surface expression markers of activation on T and B cells, and did not evaluate specific subsets of B cells that may be preferentially impaired [49]. Furthermore, some studies have demonstrated decreased ability of HIV-infected individuals B cells to differentiate toward plasmablasts in long-term culture. This has been attributed to decreased ability of T follicular helper cells (CD4+CXCR5+) to secrete IL-21 [50,51]. Our study consisted of overnight assays not designed to evaluate B cell differentiation or antibody secretion. Future studies will be designed to evaluate the specific subsets of antigen-specific T cells responsible for the activation and differentiation of B cells.

 Here we show B cell responsiveness to HIV antigen is a sensitive correlate of control of viremia. We also show that B cell responsiveness to non-specific and HIV-specific stimulation can be enhanced by the presence of CD4+ T cells and blockade of the inhibitory receptor PD-1. Understanding the interactions between CD4+ T helper cells and B cells will increase our understanding of the humoral immune response over the course of HIV infection, as well as factors which contribute to its preservation or dysfunction. A successful HIV vaccine will likely need to generate both robust cellular immune responses and broadly neutralizing antibodies [52], therefore understanding the precise interactions between CD4+ T cells and B cells will be of paramount importance. Our ongoing experiments will more specifically characterize subsets of CD4+ T cells able to efficiently stimulate B cells to differentiate into antibody-producing plasmablasts, and will help evaluate vaccines designed to elicit helper responses and neutralizing antibodies.
